# Paper and thread as media for the frugal detection of urinary tract infections (UTIs)

**DOI:** 10.1007/s00216-021-03671-3

**Published:** 2021-10-19

**Authors:** Amrutha Hasandka, Ankita Ramchandran Singh, Anusha Prabhu, Hardik Ramesh Singhal, M. S. Giri Nandagopal, Naresh Kumar Mani

**Affiliations:** 1 Microfluidics, Sensors and Diagnostics Laboratory (μSenD), Department of Biotechnology, Manipal Institute of Technology, Manipal Academy of Higher Education, Manipal, Karnataka 576104 India; 2 Department of Chemical Engineering, Manipal Institute of Technology, Manipal Academy of Higher Education, Manipal, Karnataka 576104 India; 3 Department of Mechanical Engineering, Indian Institute of Technology, Kharagpur, Kharagpur, 721302 India

**Keywords:** Urinary tract infection, Detection, Paper, Thread, Microfluidics

## Abstract

Urinary tract infections (UTIs) make up a significant proportion of the global burden of disease in vulnerable groups and tend to substantially impair the quality of life of those affected, making timely detection of UTIs a priority for public health. However, economic and societal barriers drastically reduce accessibility of traditional lab-based testing methods for critical patient groups in low-resource areas, negatively affecting their overall healthcare outcomes. As a result, cellulose-based materials such as paper and thread have garnered significant interest among researchers as substrates for so-called frugal analytical devices which leverage the material’s portability and adaptability for facile and reproducible diagnoses of UTIs. Although the field may be only in its infancy, strategies aimed at commercial penetration can appreciably increase access to more healthcare options for at-risk people. In this review, we catalogue recent advances in devices that use cellulose-based materials as the primary housing or medium for UTI detection and chart out trends in the field. We also explore different modalities employed for detection, with particular emphasis on their ability to be ported onto discreet casings such as sanitary products.

**Graphical abstract:**

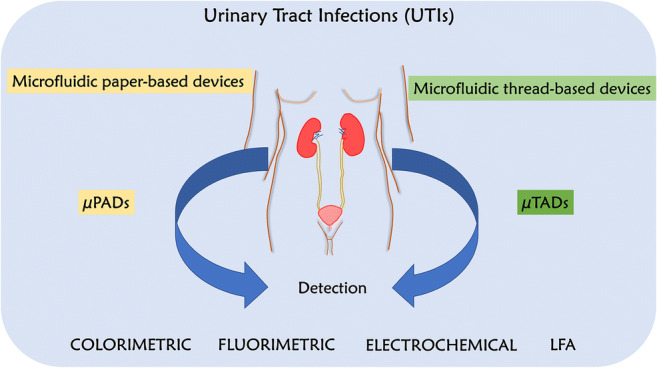

## Introduction

Urinary tract infections (UTIs) are the most prevalent type of infection observed in women and the elderly. A large proportion of the female population has endured this at least once in their lifetime, and this risk increases as women age [1]. The presence and growth of microorganisms such as bacteria and fungi in the urinary tract is the main cause of UTIs. Bacteria that reside in the urethra, vagina, and digestive tract can inadvertently lead to a UTI and can also affect areas of the body such as the lower urinary tract or the bladder [2]. The bacteria from these regions travel through the urethra and then migrate to the kidneys and the bladder [3]. Pregnant women are more highly susceptible to UTIs than men, due to many anatomical variations such as shorter urethras, relatively easy contamination of the urinary tract by bacteria from the digestive system, i.e. fecal microbiota, and many other causes [4–7]. UTIs are more frequently caused by Gram-negative organisms than their Gram-positive counterparts. Gram-negative bacteria such as *Escherichia coli*, *Klebsiella*, *Proteus*, and *Pseudomonas* spp. have been identified in UTI cases, with *E. coli* being the most common. Additionally, Gram-positive bacteria including *Streptococcus*, *Staphylococcus*, and *Enterococcus* species are know to cause UTIs [7–10].

In addition to bacteria, fungi can also be pathogenic agents of UTI [11]. Studies have revealed a decline in the percentage of UTIs caused by the commonly responsible Gram-negative organisms and a growing percentage of UTIs caused by fungi, such as *Candida* species, particularly in the case of critically immunocompromised patients [11–13]. The pervasive risk of *Candida*-based UTIs is well studied and acknowledged, including in infants, the elderly, females, patients on immunosuppressant or antiseizure medication, and those with diabetes mellitus, prolonged hospitalization, recent consumption of broad-spectrum antibiotics, past surgeries irrespective of being urological in nature, abnormalities of the urinary tract, and catheterization, among many more [14–16]. In the elderly, upper UTI can cause fever, abdominal tenderness, nausea, and vomiting. The gastrointestinal and respiratory systems of these patients can also be affected. The elderly and postmenopausal women can develop urinary urgency, painful voiding, and low-back pain, among other symptoms. Premenopausal women may also experience a burning sensation with urination [17, 18]. These clinical presentations, which illustrate the distress caused by UTIs afflicting women and the elderly, highlight the need for further research in point-of-care diagnostics for UTI detection.

**Fig. 1:**
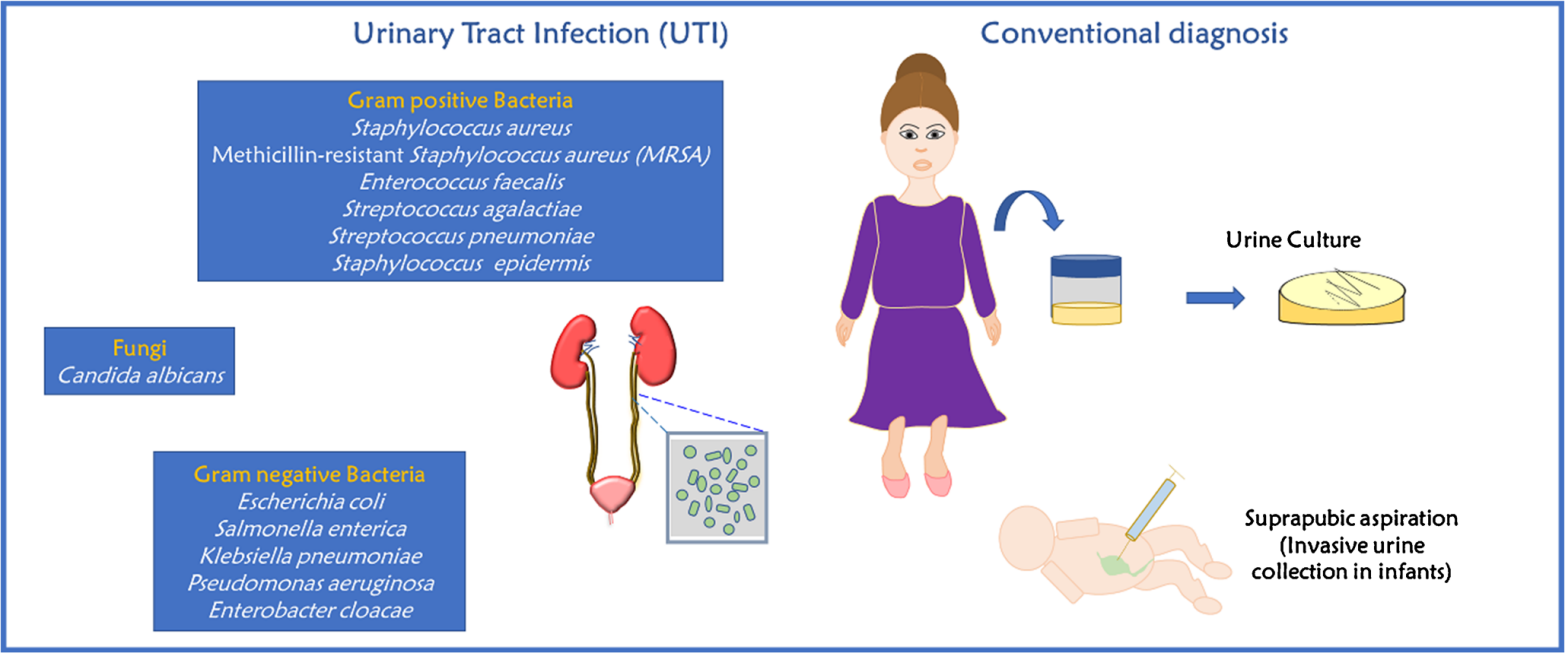
Schematic illustration of urinary tract infection with its causative pathogens and conventional diagnostic methods

The urine-based culture method is the most reliable test for determining the presence of UTI. This conventional method requires trained personnel and 48 h to confirm the presence of pathogens (bacteria or fungi) and is also labor-intensive [19] (Fig. 1). Along with urine samples, blood has also been explored as a way to ascertain the presence of pathogens [20]. In addition to culture-based methods, rapid tests using a dipstick have been investigated, where the urine was tested for analytes such as leukocyte esterase, blood, protein, and nitrites [21]. These are also efficient because they can be performed quickly and easily. Among such methods, enzymatic tests are widely used to detect bacteria in urine, or pyuria, including catalase, glucose oxidase, nitrate reductase (Greiss test), and leukocyte esterase. These tests can also be performed at home, as they are noninvasive, easy to administer, and economical, and provide rapid results. However, dipstick-based tests also have considerable trade-offs, most notable of which is their low sensitivity (<90%) [22]. Moreover, dipstick-based tests are prone to contamination by the presence of commensal bacteria picked up when passing through the distal urethra. The reactions on the dipsticks can also be complicated and may be altered by complicated analytical chemistry which may be disrupted by oxidization, reduction, and discoloring agents in the urine, making dipstick-based tests further prone to false-negative and false-positive results [19].

Enzyme-based assays can also be utilized for detecting UTIs [23] by leveraging the catalase enzyme produced by most UTI-presenting pathogens as an indicator. A catalase test can be used for detecting UTIs, but false positives can occur if the catalase is detected due to erythrocytes, white blood cells, or kidney cells. Bacteria also normally metabolize glucose in urine, so a positive glucose oxidase test could be assumed to indicate infection. However, this method is highly sensitive and hence could easily produce false-positive results as well as false negatives when performed on patients with diabetes. Nitrite present in urine has been used as an indicator of UTIs, which can be detected using the Greiss test. This test, however, has low sensitivity [24]. Microscopic examination of urine has been utilized for detecting UTI-causing pathogens via Gram staining. The collected sample is spread on a slide, air-dried, dyed using Gram stain, and then observed under a microscope. However, a notable disadvantage is that the characteristics of the test results tend to be ill-defined, since various criteria are used to determine a positive test result. It is also quite an insensitive test, showing positive results only if the bacterial concentration in the urine is ≥10^5^colony-forming units (CFU)/mL. In addition, it requires a significant amount of labor [20]. Various colorimetric [25, 26] and bioluminescence [27, 28] tests are also available; however, they are quite labor-intensive and were found to provide several false-negative and false-positive results [24].

The detection modalities discussed above present myriad testing paradigms that may be pursued for detecting UTIs. Critically, however, their utility is greatly diminished in the Global South, where they are needed the most. Therefore, the call for a more frugal design and portable porting medium has led scientists to consider readily available cellulose-based substances as potential housing candidates. For over a decade, cellulose substrates such as paper- and thread-based analytical devices (μPADs/μTADs) have been extensively developed and investigated for a wide range of applications including food and water analysis and other environmental applications [29–34]. Additionally, the biological relevance of μPADs and μTADs in blood separation and analysis, immunoassay, analyte testing, and usage of smartphones for biosensing have been studied for point-of-care diagnosis [29, 35–37]. Papers and threads are made of natural cellulose or synthesized polymer fibers. The manufacturing process allows them to be porous, with capillary gaps that facilitate the flow of liquid. Due to their low cost, portability, biocompatibility, biodegradability, and easy incorporation with other materials, these cellulose-based devices demonstrate a vast application field for fabrication of point-of-care μPADs/μTADs.

**Fig. 2:**
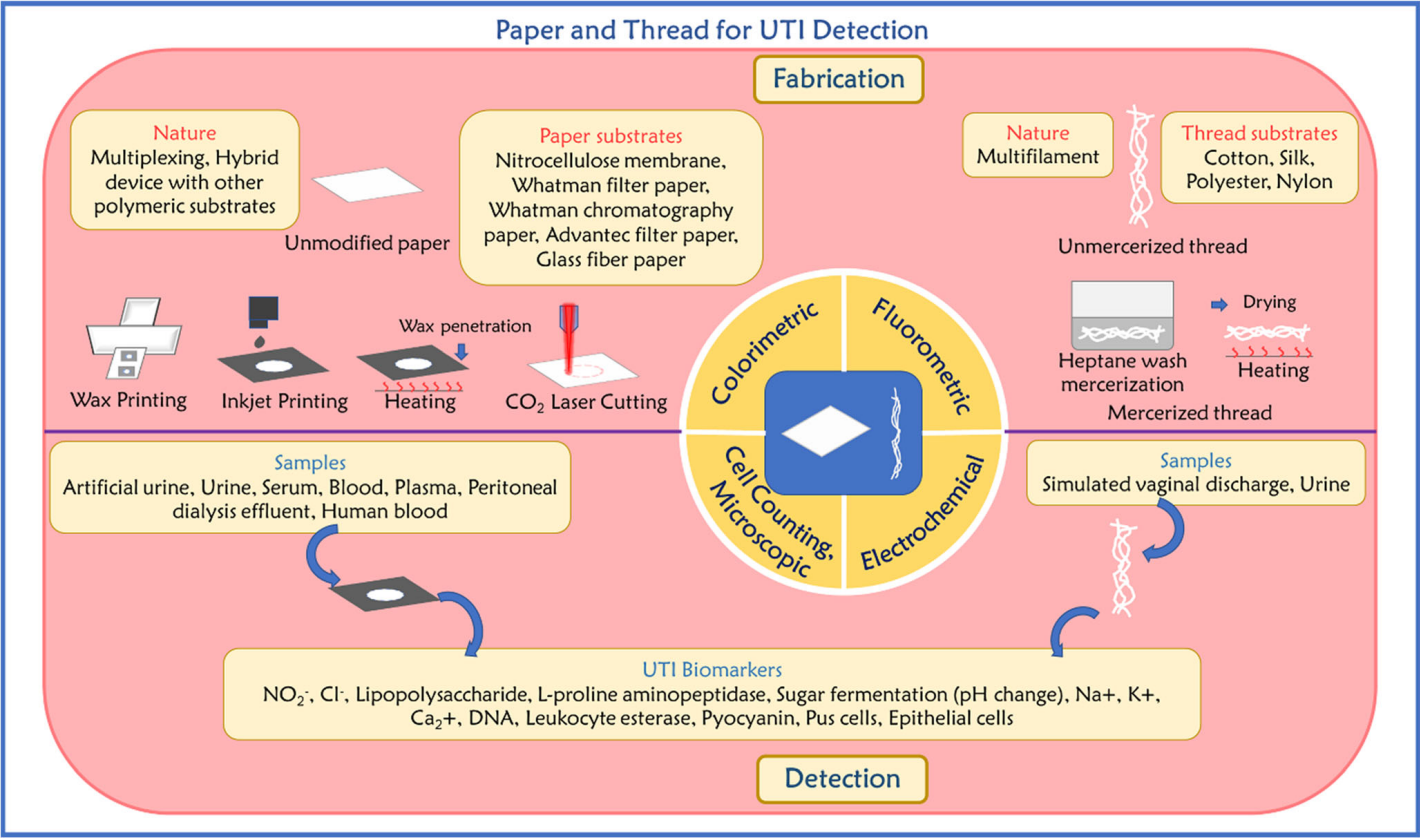
Paper and thread-based microfluidic platforms for detecting UTI pathogens and biomarkers

It can also be noted that there have been a significantly larger number of studies performed on paper than on thread devices [35, 36, 38]. Various detection methods including colorimetry, fluorometry, and electrochemistry have been utilized for these devices, making them ideal candidates for housing diagnostically relevant assays for easy use [39–41]. Along with paper, other cellulose-based substrates have also been combined with regular paper-based devices, such as introducing hemp paper alongside the commonly used filter paper. This device was explored for its potential in potassium detection in urine samples [42]. In this review, we will highlight various studies and developments which have leveraged cellulose-based substrates (paper and thread) and signal transduction methods for the detection of UTIs by targeting a range of biomarkers and pathogens in body fluids via invasive and noninvasive approaches (Fig. 2). We also discuss the possibility of integrating paper- and thread-based devices with hygiene products such as napkins, tampons, and diapers for discreet detection of UTIs.

## Detection of urinary tract infection

### Cellulose substrates as microfluidic analytical devices

Cellulose substrates, including paper and thread, have been gaining momentum as testing media in health systems, food safety, and environmental monitoring applications in light of their multifaceted properties which are conducive for low-cost fabrication and device portability. Microfluidic paper-based and thread-based analytical devices, commonly known as “μPADs” and “μTADs,” respectively, are reliable and can be widely used in performing assays and diagnostic tests without requiring large quantities of analytes, making them great candidates for remote detection of infections in humans. The use of paper, being highly flexible, porous, biodegradable, easily portable, and disposable, makes the integration of different signal transduction methods highly feasible. The efficiency by which hydrophobic barriers can be imprinted onto the PADs is a large determining factor in the overall manufacturing process. Since they are facile and economical to use, PADs can also be highly customized for a particular use case. Initially, PADs could only be used to identify one specific analyte at a time. To improve the device in this respect, multiple isolated test zones can be created on the device to allow the detection of multiple substances at once. A mechanical or chemical barrier can be used to prevent mixing to some degree. Methods for generating such barriers include photolithography, stamping, origami, and chemical modification. One method for imprinting a hydrophobic barrier on a PAD to prevent leakage of the analytes is discussed later in this section [38, 43–45].

While μPADs were developed as an alternative to overcome the limitations of conventional methods, they too are susceptible to limitations. The need for a hydrophobic barrier and their low mechanical strength and low surface tension can limit their wider application to a broad array of microfluidic analytical devices [46–49]. This led to the exploration of thread as a possible substrate for microfluidic device applications. Features such as their wide utility, high accessibility, low cost, general hydrophilic nature, and biodegradability have promoted wide interest in their potential use [50–55]. Thread-based microfluidic devices employ threads as a means for fluid retention and transport, similar to the fluid channels in μPADs, which can execute a variety of analytical assays. Commercial thread consists of thin strands of cotton, nylon, or other fibers which, when twisted together, facilitate the wicking property, or the flow of fluid without an external pumping system. Furthermore, threads tend to display superior tensile and overall mechanical strength when compared to paper, even when wet. Other advantages of μTADs include the ability of surface modulation via immobilization or inclusion of chemicals to fabricate reaction and detection zones [50] and reduce loss of solution volume due to the improved confinement of fluids [36].

Biocompatible substrates such as paper and threads of cotton and lignocellulose have recently been used increasingly frequently in the field of biosensor research as a result of their multiple beneficial use cases and low economic cost. They have already been employed in a variety of colorimetric assays for qualitative and quantitative analyses of nitrates and nitrites. Since these substances are by-products of bacterial metabolism, they can be used as analytes in samples such as urine and saliva to identify the presence of bacterial infection. One such device was utilized for environmental monitoring, where it was used to culture and identify bacteria based on T4 bacteriophage infection of *E. coli* and to confirm the presence of the enzyme β-galactosidase [56].

A marked advantage of using cellulose, particularly paper, is that researchers are able to design testing solutions that can operate in multiple functional directions [57]. The flexibility of the 3D matrix also allows complex multiplexing to be implemented rather easily by leveraging simple origami procedures [58]. The potential to test for multiple biomarkers on a single platform is particularly beneficial for UTI detection, as it enables researchers to gain more accurate analytical insight into the nature of the disease in question without having to perform multiple tests separately [59]. Furthermore, the current absence of a test that can accurately account for all UTI test cases calls for the use of a multipronged testing regime which can help build confidence in the final diagnosis [60]. Every testing scenario requires its own “best fit” morphology, which can range from lateral flow assays (LFAs) to complex 3D origami structures, each of which is discussed in detail in the sections that follow.

### Detecting UTIs using paper- and thread-based cellulose substrates

#### Colorimetric detection

Colorimetric detection owes a large portion of its appeal to its facile use and visibly differentiable endpoint. However, it can also suffer from disadvantages such as high detection limits and small linear ranges, as well as limited sensitivity [61]. Furthermore, saturation may be reached in color formation, which may also affect the accuracy of cellulose substrates like paper. Colorimetric reactions are frequently difficult to detect at low concentrations, and some sample matrices may produce a background color on the paper [62]. Despite these drawbacks, however, the many advantages and potential for improvement have led to increased research on this technique.

**Fig. 3:**
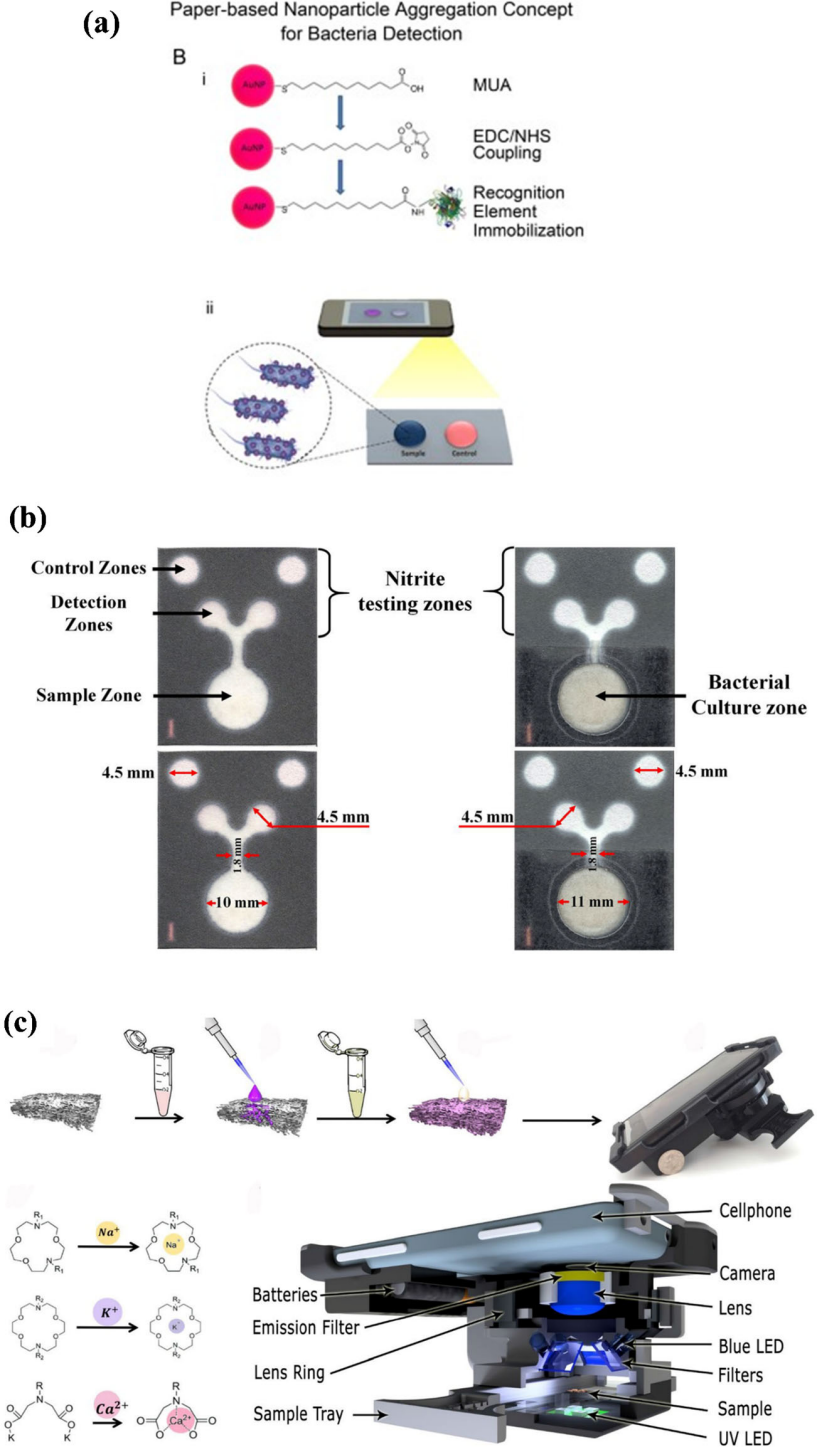
(**a**) Concept of nanoparticle aggregation for detecting bacteria using cellulose paper and smartphone [63]. (**b**) Design of a paper-based analytical device (PAD) for nitrite quantification, nitrite determination, bacterial cultivation, and identification with dimensions [65]. (**c**) Schematic illustrating the working procedure for measuring the cation concentration in samples [66]

An example of the viability of colorimetric detection in paper-based biosensors is demonstrated by Shafiee et al. [63], who developed a method that integrated cellulose paper and polyester films for use as biosensors. The device employed various detection methods using electrical/optical mechanisms incorporating antibodies and peptides for the detection of viruses and bacterial species from blood or plasma. Other analytes or by-products besides antibodies of pathogens can also be used for the detection of pathogens (Fig. 3a). Chen and Dong present a paper-based device that was fabricated using wax which was melted onto a filter paper, upon which a pattern was transferred. This micro-PAD was then leveraged for detecting nitrite, with a sensitivity range of 0.5 ppm to 100 ppm in urine samples collected in adult diapers. This could be a great low-cost, disposable UTI detection kit in remote areas [64].

Another effort in using diapers to detect biomarkers in urine was undertaken by Couto and Dong [67]. An eight-layer device was produced which can utilize a self-locking technique to close the sample inlet within 5 min. This device was able to retain test results for 8 h, which is economical and highly effective, making it a potential candidate for analysis of samples in the future.

Apart from these techniques, DNA-based detection has also been explored extensively. A signal-producing electrochemical aptamer-based sensor was reported to detect ampicillin. The signal was based on target binding changes in the methylene probe which allowed the sensor response to be given within 200 s [68]. Another study reported a device capable of cultivating and identifying bacteria in situ [65]*.* This method in particular was quicker than the usual methods for UTI detection (Fig. 3b). The cultivation-based detection method relied on β-glucuronidase detection to indirectly determine whether *E. coli* was present in the sample. The interpretation of results is fundamental for accurate diagnostic results in tests, especially when they are self-administered. Therefore, in order to increase the objectivity and user-friendliness of the devices in question, the integration of smartphones for analysis of results can also be pursued. An example of this was demonstrated in 2020 by Ghaderinezhad et al., who created a smartphone-linked device for detecting the volume of various electrolytes in urine. A colorimetric method was used to detect anions such as NO_2_^−^ and Cl^−^(Fig. 3c). This method was also shown to be potentially useful for diagnosing various kidney and-urine related disorders and has the ability to sense the daily concentration of ions, which can help identify disease in the case of abnormal numbers or spikes [66].

That same year, in 2020, an assay was reported that utilized arrays of nanoparticles to detect various bacterial species which could potentially detect UTIs. This system worked by monitoring the reaction between bacterial metabolites and the nanoparticles on a paper-based substrate. The color changes were unique to different strains of bacteria, and even very low concentrations could be detected in minutes, which is extremely fast. Additionally, thread has been leveraged as a detection device used for pathogen identification and quantification. A study by our lab used multifilament cotton thread in a microbial detection assay with smartphone imaging for quick pathogen detection. The detection was based on the by-products released by the pathogens as a result of the metabolic breakdown of the different sugars in the media in question. This method was highly scalable and could potentially differentiate between different attacking pathogens [69] (Table 1).

**Table 1:** Urinary Tract Infection causing pathogens and detection mechanisms

Fabrication	Salient materials	Device structure	Pathogen/biomarker/analyte	Sample	Detection mode	Limit of detection	Ref
Wax printing	Whatman no. 1 filter paper, Griess reagent (nitrite assay)	Y-shaped channel with detection zones at two ends and a sample zone at one end	*Escherichia coli* (nitrite)	Spiked control urine	Colorimetric	10^4^ CFU/mL	[65]
–	Whatman No. 1 filter paper, chloride assay kit. sulfanilamide, N-(1-Naphthyl) ethylenediamine and citric acid (Nitrite detection), smart-phone	–	NO_2_^-^, Cl^-^	Artificial urine	Colorimetric	0.13 mM (NO_2_^-^)	[66]
Wax printing	Acrylic base, wooden chopstick as rotation axis and three layers of Whatman filter paper, biotin, HRP, anti-*E. coli*	Turntable with six reagent zones	*E. coli (anti-E. coli)*	*E. coli* in Luria-Bertani (LB) medium	Colorimetric	10^5^ CFU/ml	[70]
Bee wax screen printing	Kraft paper, acrylate, Poly (methyl methacrylate) (PMMA), polytetrafluoroethylene (PTFE), filter paper	Set of eight layers one on top of the other, Test layers made of filter paper cut in a parallelepiped shape, reagent pads glued between wax barriers by adhesives	Glucose, protein, occult blood, nitrite, leukocyte esterase	Urine	Colorimetric	–	[67]
CO_2_ laser cutting,	Whatman filter paper, solid wax, PMMA	Star patterned paper with hydrophobic barriers and hydrophilic channels connected with eight reagent zones	Nitrite	Spiked urine	Colorimetric	0.5 ppm	[64]
CO_2_ laser cutting	Whatman filter No.1, specific aptamer for *S. aureus*, Au/Pt bimetallic nanocluster, TMB	Y shaped channel with one end for control and another for the target	*Staphylococcus aureus*	Human serum	Colorimetric	80 CFU/mL	[71]
Inkjet printing of Paraffin ink	Whatman filter paper (grade 1), colorimetric substrate PRO and indicator DCA	Circular device	*Candida albicans* (L-proline aminopeptidase)	Spiked water	Colorimetric	0.86 × 10^6^ CFU/mL	[72]
–	Whatman 1 filter paper, Whatman 1 chromatography paper, resazurin sodium salt, smart-phone	Test strips	*Enterococcus faecalis*	*E. faecalis* in brain heart infusion (BHI) broth	Colorimetric	–	[73
Computer-numerical-control (CNC) machine process	PMMA, nitrocellulose membrane loaded with aptamers and reaction buffers, ashless filter paper	Four PMMA layers -the cover, support layers I and II, and the reservoir chambers, a nitrocellulose membrane, and a support paper all placed one over the other	*E. coli,* Methicillin-resistant *Staphylococcus aureus*	Spiked Trypticase soy broth with yeast extract, Spiked joint fluid	Colorimetric	10^4^ and 10^5^ CFU/μL	[74]
–	Cellulose paper, modified gold nanoparticles with lipopolysaccharide binding protein (LBP) for *E. coli* and anti-LTA for *S. aureus*	–	*E. coli* (Lipopolysaccharide) *S. aureus* LTA)	Spiked whole blood, plasma and peritoneal dialysis effluent	Colorimetric	8 CFU /ml	[75]
Mercerization with heptane wash	Twisted multifilament cotton thread (TMC), piping white glazed cotton thread (PWGC), Sanitary napkins, tampons, Colorimetric substrate PRO and indicator DCA	Embedded substrate-imbibed TMC and PWGC thread in the inner layer of sanitary napkins and tampons	*C. albicans* (L-proline aminopeptidase)	Simulated vaginal discharge	Colorimetric	0.58 × 10^6^ CFU/mL	[76]
–	Twisted multifilament polyester thread (TMPT), cotton knitting and hand sewing yarn (CKAHSY), best quality twisted polyester yarn (BQTPY) and twisted multifilament cotton thread (TMC), phenol red dye, smart-phone	–	*C. albicans, E. coli* (pH)	Spiked phosphate buffered saline	Colorimetric	–	[69]
CO_2_ laser cutting	Chromatography paper (Whatman no. 1), fluorescent probes Sodium Green, PBFI potassium-sensitive dye and Fluozin calcium indicator, smart-phone, emission filters, lens, Blue and UV LEDs	–	Na+, K+, Ca_2_+,	Artificial urine	Fluorometric	1.26, 0.85,1.2 mM	[66]
Lithography	Cellulose chromatography paper, Anti-*E. coli* antibody and BSA conjugated polystyrene latex particles, smart-phone	Multiple channels consisting of a straight channel (2.5 mm × 11.5 mm), including an area of detection and an oval shape for an absorbent pad (4.5 mm × 5.5 mm)	*E. coli*	Spiked urine	Fluorometric	10 CFU/mL	[77]
Wax printing	Loop-mediated isothermal amplification (LAMP) reagents, biotinylated primer, cellulose membrane paper, DNA fluorescent dye	Sandwich device consisting of two pieces of double-sided adhesive tape (2 mm thickness) as the bottom base, wax-coated cellulose membrane paper as the reaction pad in the center and a clear top seal	Methicillin-resistant *S. aureus*	Spiked whole blood	Fluorometric	10 ag (1 copy of MRSA gene *mecA*)	[78
Wax printing	Polyether sulfone, glass fiber, cellulose acetate, absorbent pad (cellulose fiber), HNB	Four circular channels. A smaller circle in the center for the sample injection hole. Reaction pads were placed on each channel of the fluidic channel pad.	*Streptococcus agalactiae, Streptococcus pneumoniae* *S. aureus* (LAMP-HNB signal)	Extracted DNA	Fluorometric	4.1 × 10^2^ copies	[79]
–	Whatman chromatography paper, PDMS, pathogen specific aptamers	PDMS top and middle layers and paper and a glass plate as the bottom layer	*S. aureus* *Salmonella enterica*	Spiked urine	Fluorometric	11.0 CFU/ mL for L. Acidophilus	[80]
Laser cutting	Cotton, nylon and silk threads, PMMA base, TRIS/CHES buffer solution, SYTO- 9	3D printed fluidic platform, set of 8 printed reservoirs. Threads go from the front part along the lined space, underneath the rolls and tied in the ring at the back side.	*E. coli*	Spiked urine	Fluorometric	–	[81]
Wax printing	Advantec filter paper (no. 5C), 3-(*N*-tosyl-l-alaninyloxy)-5-phenylpyrrole (PE) and 1-diazo-2-naphthol-4-sulfonic acid (DAS)	–	Leukocyte esterase (DAS)	Urine	Electrochemical	1.91 × 5.1 U mg^-1^ mL^-1^	[82]
Wax printing	Filter paper, 2B graphite leads, 3 M blue tape, conductive silver ink	Device of dimension 1.8cm × 2cm including electrode regions and 12mm × 8mm sample/reagent zone.	*Pseudomonas aeruginosa* (pyocyanin)	Spiked saliva	Electrochemical	10 nmol L^−1^	[83]
–	Whatman no. 3, pore grid filter, white and black grid, liquid media	Three-layer, circular filter paper sandwich applied to the surface of petri dish	E. coli, S. aureus, Staphylococcus epidermidis, E. faecalis, C. albicans, Klebsiella pneumoniae, Enterobacter cloacae	Urine	Cell counting	≥ 10^4^ CFU/ml Gram-negative bacteria	[84]
–	–	Paper-based Foldscope	Pus cells, epithelial cells	Urine	Microscopic	–	[85]

A method demonstrating paper-based enzyme-linked immunosorbent assay (ELISA) for rapid detection of *E. coli* was also reported. While this method does have several steps in the procedure, it was successfully used to detect *E. coli* when it was present at a concentration of under 10^5^ CFU/mL. If improved upon, these could potentially be used for at-home detection kits [70]. In another study, a soaking-drying method was implemented on a resazurin-deposited PAD for the rapid detection of bacterial species and their biotoxicity. A color change occurred due to the reduction of the resazurin by the metabolic processes of *Enterococcus faecalis.* This could also be recorded and analyzed by smartphones [73].

#### Fluorometric detection

Fluorometric methods are frequently reported and generally have benefits over traditional biochemical assays, including lower cost and high sensitivity and reproducibility, which are further supplemented by the introduction of smartphone-based analysis [86, 87]. Conventional methods for conducting fluorescence assays in point-of-care diagnostics often use specialized electronic devices such as fluorescence microscopes, scanners, and other equipment that may be too resource-intensive for use in the field or low-resource environments [88].

Therefore, fluorometric detection which can be performed on paper-based devices is essential for the detection of UTIs in the Global South. To this end, a polydimethylsiloxane(PDMS)/paper/glass hybrid microfluidic device was developed with aptamer-functionalized graphene oxide (GO)nano-biosensors [80] for the detection of various pathogens [80]. Paper was utilized as a substrate to avoid complex surface treatment, and the aptamer was adsorbed on the GO surface in order to quench the fluorescence. The presence of the pathogens aided in the release of the aptamer, thus restoring its fluorescence signal. The pathogens that were used for detection were *Staphylococcus aureus* and *Salmonella enterica*. However, *Lactobacillus acidophilus* was the bacterium employed to determine the detection limit of 11.0 CFU.

**Fig. 4:**
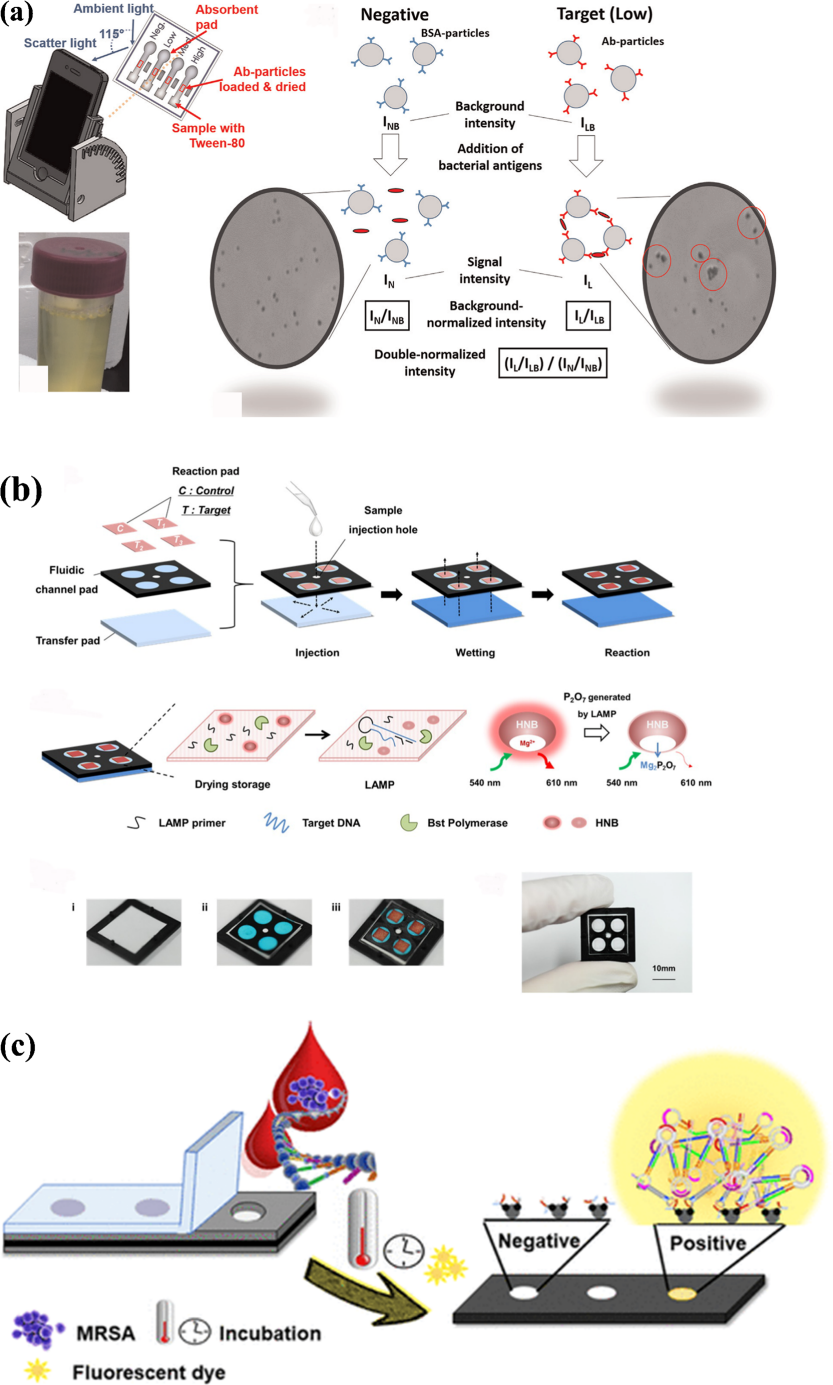
(**a**) Schematic for the smartphone-based, sensitive μPAD detection of UTI gonorrhea. Reprinted with permission from Ref. [77]. Copyright 2015 Elsevier. (**b**) Schematic representation of a paper-based device for performing loop-mediated isothermal amplification (LAMP) with real-time simultaneous detection of multiple DNA targets [79]. (**c**) Loop-mediated isothermal amplification of methicillin-resistant*Staphylococcus aureus* using paper devices and further validated with clinical samples. Reprinted with permission from Ref. [78]. Copyright 2021 American Chemical Society

Antibody-based fluorometric detection methods have also been utilized in UTI pathogen detection. For example, a paper-based device was reported for identifying UTIs, with *E. coli* as the pathogen, as well as for identifying sexually transmitted diseases (STDs) such as gonorrhea from human urine samples [77]. Anti-*E. coli* or anti-*Neisseria gonorrhoeae* antibodies were conjugated onto submicron particles, then pre-loaded and dried on the center of a paper microfluidic channel, which was then used for the pathogen detection (Fig. 4a). An additional benefit of the paper housing was the filtration of possible false-positive signal-causing agent urobilin, responsible for the yellow appearance of urine, along with green fluorescence emission by the paper-based device. Additionally, immunoagglutination of antibody-conjugated particles such as anti-*E. coli* were formed and quantified via smartphone camera detection. The limit of detection established was 10 CFU/mL. In addition to paper-based devices, thread-based devices have also been employed in pathogen or pathogen-biomarker isolation. An interesting thread-based device on a 3D printed platform was designed and fabricated by Cabot et al. to separate charged solutes, biomolecules, and intact bacterial cells [81]. Threads of different types of fibers were knotted onto each other and used electrophoretically to isolate target solutes or biomolecules, after which various fluorescence reagents were utilized for signal detection of the target particles. *Escherichia coli* was used as a pathogen to test the application of live bacteria detection from spiked urine samples.

Just as in colorimetric methods, DNA-based detection has also been a useful technique for pathogen identification when integrated with fluorescence-based detection. A paper-based device capable of detecting multiple DNA targets through loop-mediated isothermal amplification (LAMP) was reported [79]. Hydroxynaphthol blue fluorescence was employed to analyze real-time LAMP signals in the paper device. *Streptococcus agalactiae*, *Streptococcus pneumoniae*, and *S. aureus* were simultaneously detected using this device, with 4.1 × 10^2^ copies of extracted DNA being the limit of detection (Fig. 4b). Other analytes or biomarkers of pathogens were also used for fluorometric analysis in a study by Ghaderinezhad et al., where a paper-based sensor was devised and created for measuring the concentration of sodium, potassium, and calcium in urine, quantified using fluorescent probes and a smartphone, for image capture and analysis [66], resulting in detection limits of 1.26 mM, 0.85 mM, and 1.2 Mm for sodium, potassium, and calcium, respectively.

More recently, Choopara et al. produced a fluorometric paper-based LAMP on a cellulose membrane paper that demonstrated accuracy similar to quantitative polymerase chain reaction (qPCR) methods (Fig. 4c). The device enabled instant detection of methicillin-resistant*S. aureus*(MRSA) from clinical blood samples using a paper strip method, in which the lowest recorded detection of the MRSA gene *mecA* was 10 ag, which is equivalent to one copy of the gene [78] (Table 1).

#### Electrochemical detection

Colorimetric approaches are effective at intuitively indicating the presence of pathogens both qualitatively and quantitatively [57]. However, colorimetric detection can be rendered less effective when dealing with certain molecules and lower concentrations. As a means to rectify this shortcoming, an appropriate electrochemical technique can be deployed to significantly improve the specificity and accuracy of an assay [89]. In this vein, Prof. Henry’s group proposed electrochemical detection as an alternative to colorimetric detection on paper-based devices [89], which can produce results with superior sensitivity and specificity. This quantitative detection was accomplished through appropriate selection of the electrochemical technique, electrode material, and electrode potential [90], while also facilitating the development of reagent-free detection applications [91]. Moreover, electrochemical methods allow for high-throughput testing, which is especially useful when analyzing large samples in point-of-care diagnostics [61]. While the need for a reference electrode and its sensitivity to pH might be potential drawbacks, various other advantages make this method appealing in the diagnostic space.

**Fig. 5:**
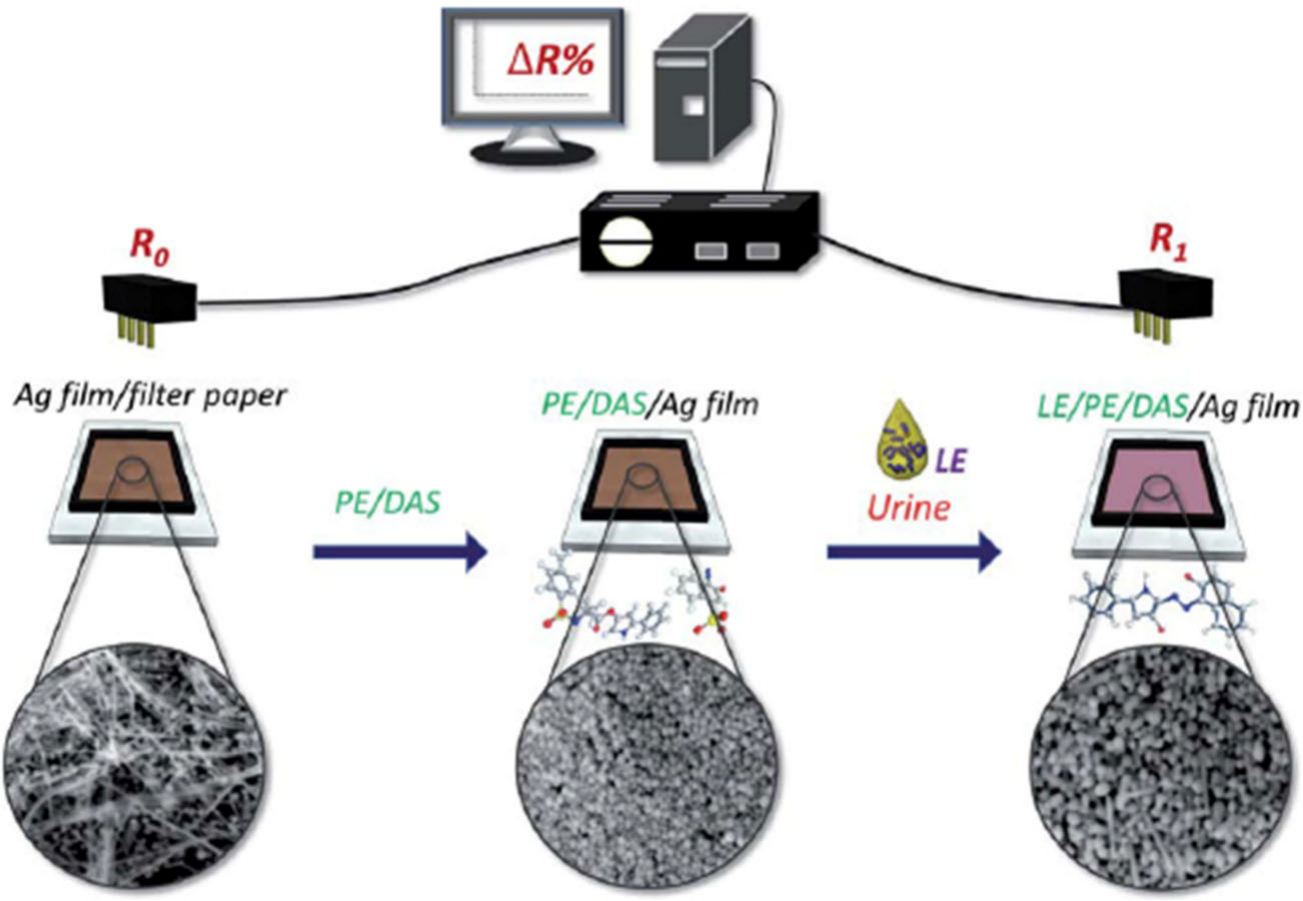
Schematic representation of leukocyte esterase-PADs for leukocyte esterase detection [82]

A paper-based analytical device that used electrochemical methods to detect the biomarker leukocyte was reported by Ho et al., which could indicate the presence of UTI from urine [82]. Various reagents were deposited onto a silver conducting film on which the leukocyte esterase from the urine reacts with the reagents, leading to a change in resistivity which is then correlated with the quantification of the target (Fig. 5). The device showed promising results regarding UTI diagnosis, demonstrating its reliability by boasting a limit of detection of 1.91 x (5.1 U mg^−1^ mL^−1^). Another team of researchers created an electrochemical paper-based device (ePAD) for the detection of *Pseudomonas aeruginosa* in human saliva. A major advantage was the lack of any sample preparation or separation procedures, which would be of great importance if ported onto a potential UTI test. Pyocyanin was the biomarker virulence factor that was used to detect the pathogen, with a detection limit of 10 nmol L^−1^ [83] (Table 1).

#### Other methods: cell counting and microscopy

Cellulose-based materials not only provide great utility as substrates for microfluidic testing, but also provide considerable advantages as housing for other, more traditional auxiliary testing modalities. A most relevant example of this is demonstrated by the use of filter paper as a solid-phase dilution device [84]. Filtration dilutes and spreads the inoculum onto a solid culture surface, which is optimized to ascertain microbial permeability through filter papers, inoculum size, and ability to exclude vaginal epithelial cells. The pathogens were inoculated and incubated on a grid to aid in colony counting. This technique was devised as a better alternative to the streak plate method. *Escherichia coli*, *S. aureus*, *Staphylococcus epidermidis*, *Enterococcus faecalis*, *Candida albicans*, *Klebsiella pneumonia*, and *Enterobacter cloacae* were among the numerous pathogens tested from urine samples that could be detected through this technique. The lowest number of cells that could form enough colonies to be considered as testing positive for infection caused by Gram-negative bacteria was ≥104 CFU/mL.

A much more rudimentary yet fundamental application of paper is its use for providing structural support for microscopy implements. Perhaps the most acclaimed demonstration of paper versatility is the Foldscope. The Foldscope is an innovative low-cost and simple paper-based microscope that can be attached to a smartphone camera for viewing. Leveraging this low-cost yet powerful analytical tool, researchers have assessed the ability of the Foldscope in the clinical diagnosis of oral and urinary tract infections [85]. Twenty-five urine samples of patients aged 11–62 years were analyzed using the Foldscope, providing a clear distinction between those who tested positive for UTIs and those who tested negative. This technique can be considered a highly valuable and inexpensive method for detecting UTIs as well as kidney stones in urine samples (Table 1).

The detection methods described provide adequate utility individually but can be specifically tailored to their required application for maximized accuracy. Colorimetric approaches, in general, tend to be the most facile and economical diagnostic approach in all cellulose-based media, as they require minimal training and equipment. Fluorometric approaches have similarly unique applications but with added complexity, as they require specialized equipment for endpoint detection. However, thread-based detection devices in particular need to implement additional calibration when used in fluorescence detection methodologies, as the natural fluorescence of the thread may induce error in the final results [92]. Electrochemical approaches, if applicable, can be adopted as an addition to the previous testing regimes in instances of lower concentration, albeit with increased operational complexity. Other modalities, similar to those previously discussed, have particular use cases but are nevertheless still more economical than most traditional methods.

## Lateral flow assay for detecting UTIs

The introduction of lateral flow assays (LFAs) to the market dates back to the early 1990s [93]. Although not directly related to cellulose-based μPADs and μTADs, their contribution to point-of-care diagnostics is especially noteworthy. The inception of the “dipstick” was revolutionary for women’s reproductive health due to its various applications such as pregnancy tests [94, 95], UTI tests [96, 97], and a multitude of other urinalysis techniques including, more broadly, diabetes/ketosis tests [98]. Their ease of use, portability, and low cost make such devices an accessible and frugal alternative to conventional diagnostic methods. Conventional lateral flow assays consist of a multifunctional array of components that overlap with one another. These components are installed onto a backing card/paper for support and fixed in place by an adhesive substance. Testing zones are constructed by creating segments of different materials such that when the urine is drawn through by capillary action, it encounters immobilized conjugates that aid in the detection of the analyte through a colorimetric readout [93].

UTIs in particular have been extensively diagnosed using devices employing lateral flow assays. While earlier studies on urine dipsticks indicated optimistic results [99], subsequent studies demonstrated their limitations such as their general low sensitivity [20, 100], and particular inaccuracies in cases of uncomplicated UTIs [101] and catheterized patients [102]. Similar results were found in a study of UTIs among pregnant women in Tanzania, where it was determined that the dipstick alone was not sufficiently reliable for accurate diagnosis [103]. These limitations motivated efforts for enhanced sensitivity. In 2015, an improvement was made in the sensitivity of an existing rapid urine-based circulating anodic antigen (CAA) test for diagnosing schistosomiasis, by increasing sample input. For many years, the preferred method had been the detection of eggs in urine. However, the detection of a worm-regurgitated antigen (circulating cathodic antigen, CCA) was observed to be comparable or even superior to the egg counting method, thus leading to this study, which was successful in improving sensitivity [104] . That same year, the rapid detection of bacteriuria was studied using an antibody-based lateral flow assay (RapidBac). Rigorous tests were conducted to determine the sensitivity and specificity of the device, ultimately concluding that the RapidBac test for bacteriuria may be valuable in emergency and primary care settings as UTI point-of-care diagnostics [105].

More recently, LFAs and dipstick testing have also been used in combination with flow cytometry for prediction models, such as deducing a probability prediction formula to diagnose a specific pathogenic strain responsible for UTIs in real time [106]. Developments in LFA technology and the subsequent commercialization of the technique [96, 97, 107] have greatly increased the accessibility to accurate UTI testing in point-of-care applications. Therefore, the development of market-ready LFA products which are leveraged towards UTI detection will remain relevant in the women’s health space (Table 2).

**Table 2:** Lateral flow assays in the detection of UTIs

Pathogen/Biomarker/Analyte	Sample	Detection Mode	LOD	Ref
Leukocyte esterase	Urine	Colorimetric (dipstick)	–	[99]
*Escherichia coli, Enteroacter cloacae, Pseudomonas* spp., *Proteus spp. Klebsiella* spp., *Staphylococcus aureus, staphylococci, Enterococcus* spp., and *Candida* spp.	Urine	Colorimetric (dipstick) and cell culture	–	[102]
Leukocyte esterase and nitrite	Urine	Colorimetric (dipstick)	–	[101]
Leukocyte esterase, nitrite, blood trace	Urine	Colorimetric (dipstick)	–	[100]
*E. coli, Enterococcus* spp., *Klebsiella pneumoniae Acinetobacter* spp., *Pseudomonas* spp., *Morganella* spp., *S. aureus, Streptococci* and *Enterobacter* spp.	Urine	Colorimetric (dipstick) and cell culture	–	[103
*E. coli, Staphylococcus saprophyticus* (monoclonal antibodies)	Urine	Colorimetric	–	[105]
Schistosomes (worm circulating anodic antigen (CAA))	Urine		10 pg/mL to 0.03 pg/mL when increasing urine sample input from 10 μL to 7.5 mL	[104]
*Enterobacter, Klebsiella, Acinetobacter* (nitrate)	Urine	Colorimteric (dipstick) and cell culture	–	[108]
*P. mirabilis* and other bacilli (proteins, ketones and nitrites)	Urine	Colorimetric (dipstick) and flow cytometry	–	[106]

## Future trends and challenges

Accessible and discreet UTI detection provides tremendous self-reliance to groups that are unable to access critical resources for traditional methods. As a result, the development of techniques that can advance personal capability in the detection of clinically relevant diseases is seeing significant momentum [109, 110]. In particular, the use of cellulose-based substrates for solving structural and sample manipulation problems is generating considerable interest, as it allows for much easier and more economical fabrication of devices [111]. The frugality of cellulose-based diagnostic media is most clearly seen in their ability to serve as an effective base onto which existing assays can be ported. The devices can house multiple stages involved in the detection process, from pathogen sampling to biomarker analysis, and their ability to operate with lower volumes of analyte greatly increases the viability of the method, as it caters to regions with less developed infrastructure, something generally absent in traditional methods. Additionally, cellulose-based materials, when used to structurally support essential equipment such as microscopes, further facilitates frugal innovation in the diagnosis of UTIs. The cost of operation can also be lowered by ensuring significant penetration of these techniques into general commercial use through incorporation into industrial and academic general practice fabrication routines. Mass adoption of the technique as previously described can be achieved by supplementing growth through the implementation of our proposed dual-pronged approach.

**Fig. 6:**
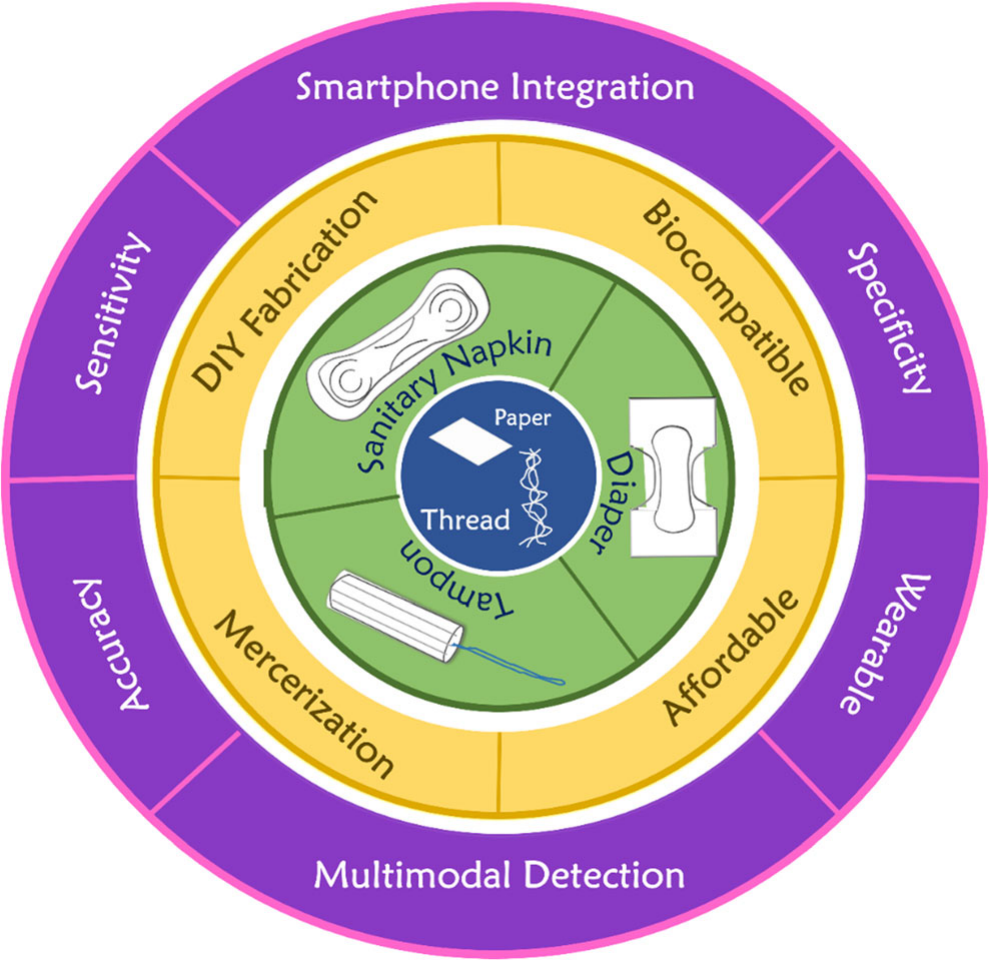
Integration of paper- and thread-based microfluidic devices with hygiene products and their possibilities

The first part of such approach would be focused on improving the base utility of the fabrication substrates by enhancing their technical robustness. In laboratory settings, progressive improvement in the performance efficiency of cellulose devices can be realized by improving the structural integrity and wicking ability of the substrate. This can be done, for example, by developing mercerization techniques geared towards increasing the water absorption and tensile strength of thread-based devices [112]. Traditional mercerization techniques typically use caustic substances such as hydrogen peroxide [113] and hydrochloric acid [114], which tend to leave residues on the substrate and can therefore interfere with the results of a thread-based test which may be sensitive to pH [115]. Additionally, traditional mercerization, in general, is a very cumbersome process that requires numerous resource-intensive treatment steps. As a result, developing long-term solutions and alternatives to these methods is essential for their utility to bear fruit. A viable alternative to this would be using heptane as a mercerizing agent, which can show similar effectiveness without adding extra heating steps and leaving any residue on the thread [76]. These methods when scaled can help produce a more suitable raw product that can be integrated into analytical devices (Fig. 6).

Another development that can contribute to achieving the proliferation of general production is the availability of a standardized manufacturing toolkit. Standardizing production on an industrial scale will require new techniques for facile fabrication which can be automated to boost the production of devices so that they are widely available to people in low-resource areas [116–118]. Standardizing an industrial toolkit can also help prevent issues with assay adaptation to the cellulose medium. Due to the complexity of the different methods and approaches for manufacturing cellulose-based analytical devices, their wider-scale adoption and use in industrial settings have been difficult [111]. If a standard toolkit is developed, a modular processing approach can be achieved, where preprocessing or postprocessing steps can be added according to the application. This method can also help in standardizing the equipment one might use for scale-up and automation, further increasing the method’s market penetration. Both these improvements can spur the development of numerous multiplexed detection devices which can detect multiple relevant diseases on a single substrate and sample, significantly increasing the efficiency of the medium [111, 119]. Facile fabrication can also possibly extend to localizing the production of analytical devices in the communities in which they are needed the most, by focusing on the do-it-yourself(DIY) aspect of fabrication [43, 120, 121]. This can be facilitated by developing techniques that use off-the-shelf materials such as pens [122], stamps [123], and general-purpose stationery [124, 125] for fabrication which can be easily done by hand or easily automated.

The second approach towards increasing the potential of cellulose-based substrates would be by lowering the barrier of entry into the use of the technology for all major stakeholders, particularly women (Fig. 6). Keeping the sustainable development goals of gender equality and reducing inequality as benchmarks [126], one can work towards increasing access to UTI detection technologies by enhancing the testing capabilities of paper-based devices and by lowering the cost of manufacturing each testing unit. As mentioned previously in this review, integrating testing devices into hygiene products can greatly increase the number of people the technology can reach and enhance its testing capabilities [76]. This can be built upon by developing additional wearable technologies which allow for continuous monitoring of relevant biomarkers, adding to the amount of data available to make informed clinical decisions [55, 127, 128]. Potential hurdles to the widespread use of wearable technology, namely the limited biocompatibility of the testing reagents and the inability of the device to be disposed of without pretreatment [129], could both be solved by using materials and chemicals that are designed with human contact in mind. Lack of particular preprocessing steps could also be a potential setback in the development of wearable cellulose-based devices [129], although further research into integrating electrochemical methods can work towards ameliorating the issue [130].

Although the current literature has limited if any examples of wearable UTI detection devices [76, 131, 132], wearable systems which use urine [133–136] or saliva [137–139] for testing can be adapted for similar use. The current boom in computing can also be exploited to greatly improve the sensitivity and specificity of testing by using smartphones for DIY image processing of the final result [66, 77]. The widespread availability and viability of smartphones allow for the development of specific applications geared towards testing socially relevant diseases [140]. Particularly important is their utility in eliminating subjectivity in the final results of an assay and their ability to directly parse electrical signals into readable data. Integrating cellulose-based devices into a smartphone-based testing regime can enable reusable and economical test sample collection which can be sourced individually for use.

Moreover, applying artificial intelligence (AI)-enabled technologies could not only increase the accuracy of the final diagnosis, but could also provide quick access to relevant medical records for future medical consultations [127]. This bridging between the digital and analog also spurs the development of personalized treatment for each patient which can be availed from anywhere around the world [141]. Testing capabilities can also be increased by incorporating multiple testing modalities on a single device, facilitating the creating of a veritable “one-stop shop” which can test numerous relevant biomarkers for multiple UTIs at the same time [110, 119, 142]. This personal testing method will favor quick and personal testing akin to pregnancy tests, which can significantly reduce the logistical hurdles individuals face in accessing testing for UTIs (Fig. 6).

Cellulose-based systems offer unique pathways towards multimodal detection of UTI infections and can supplement current diagnostic systems greatly. Nevertheless, it is prudent to note that cellulose-based diagnostic devices are also subject to complications inherent in UTI detection. Given the relatively rudimentary nature of the diagnostic devices, most of the devices discussed in the literature rely on the presence of pathogens in urine as a means of diagnosis. Detection in this manner has low diagnostic accuracy and clinical utility, as the data might change according to the stage of infection and can also often be conflated with results present in asymptomatic bacteriuria, which in most cases does not require intervention [60, 143, 144]. Another sizable part of the literature focuses on using conventional biomarkers such as urine nitrite and leukocyte esterase. These biomarkers have been shown to be effective in most clinical settings and can be used to diagnose UTIs in different stages of infection, but generally also have low sensitivity and specificity for predicting or differentiating UTIs [60, 144, 145]. Novel and more accurate biomarkers like interleukins [60] and lactoferrin [146] are yet to be explored in this medium. Additionally, complex detection methods such as spectrometry [147] are harder to port onto a cellulose-based medium. However, the absence of a biomarker that can be independently used to check for UTIs can be uniquely dealt with using multiplexed cellulose-based devices, as they can help significantly with the cost-effective testing of multiple biomarkers simultaneously.

In this review, we have highlighted the many developments in the field of cellulose-based substrate testing of UTIs and the utility they find in many clinical applications. Through review, one can see that the technology, although still in its infancy regarding commercial use, has tremendous potential as a medium to bridge the gap between the development of testing techniques in the lab and their introduction in the general market. Despite their many advantages, we envisage these testing methods not as a way to eclipse the use of standard testing but as a supplement to laboratory testing, offering multiple testing modalities for UTI diagnoses. These methods should command more attention, as they push the limit of what is considered feasible to achieve when leveraging low-cost materials towards high-level diagnostics and can pave the way towards many new exciting developments in the field of analytical devices.
